# Measuring Mitochondrial Oxygen Tension during Red Blood Cell Transfusion in Chronic Anemia Patients: A Pilot Study

**DOI:** 10.3390/biomedicines11071873

**Published:** 2023-06-30

**Authors:** Rinse Ubbink, Lucia W. J. M. Streng, Nicolaas J. H. Raat, Floor A. Harms, Peter A. W. te Boekhorst, Robert J. Stolker, Egbert G. Mik

**Affiliations:** 1 Laboratory of Experimental Anesthesiology, Department of Anesthesiology, Erasmus MC, University Medical Center Rotterdam, 3000 CA Rotterdam, The Netherlands; 2 Department of Hematology, Erasmus MC, University Medical Center Rotterdam, 3000 CA Rotterdam, The Netherlands

**Keywords:** mitochondrial oxygen tension, mitochondrial oxygenation, red blood cell transfusion, chronic anemia

## Abstract

In light of the associated risks, the question has been raised whether the decision to give a blood transfusion should solely be based on the hemoglobin level. As mitochondria are the final destination of oxygen transport, mitochondrial parameters are suggested to be of added value. The aims of this pilot study were to investigate the effect of a red blood cell transfusion on mitochondrial oxygenation as measured by the COMET device in chronic anemia patients and to explore the clinical usability of the COMET monitor in blood transfusion treatments, especially the feasibility of performing measurements in an outpatient setting. To correct the effect of volume load on mitochondrial oxygenation, a red blood cell transfusion and a saline infusion were given in random order. In total, 21 patients were included, and this resulted in 31 observations. If patients participated twice, the order of infusion was reversed. In both the measurements wherein a blood transfusion was given first and wherein 500 mL of 0.9% saline was given first, the median mitochondrial oxygen tension decreased after red blood cell transfusion. The results of this study have strengthened the need for further research into the effect of blood transfusion tissue oxygenation and the potential role of mitochondrial parameters herein.

## 1. Introduction

Since 98% of oxygen in the blood is bound by hemoglobin (Hb), red blood cells play a vital role in oxygen transport from the alveoli in the lungs to tissues in the entire body [1]. In cells, mitochondria use oxygen for oxidative phosphorylation to create adenosine triphosphate (ATP). This oxygen-dependent process is highly efficient in generating ATP. In comparison, anaerobic glycolysis results in 88% less ATP per glucose molecule [2]. Therefore, a reduced level of red blood cells (RBCs), or anemia, compromises metabolic capacity and the processes depending upon it.

The cornerstone of treatment for anemia is a red blood cell transfusion (RBCT). However, an RBCT has various risks. These may involve blood-transmitted bacterial or viral pathogens, immunological reactions, such as an acute hemolytic reaction or acute lung injury related to transfusions (TRALI), hemodynamic or electrolyte disorders, or simply administration of the wrong product through human error [3]. It follows that RBCTs should only be given to patients for whom the benefits outweigh the risks [4].

Currently, the decision to transfuse erythrocytes in most clinical cases is mainly based on a level of hemoglobin specified as a transfusion trigger. To prevent unnecessary RBCTs, studies have compared the effects of transfusion trigger levels, with particular attention to differences between liberal (5.6 mmol/L to 6.21 mmol/L) or restrictive (3.34 mmol/L) transfusion triggers [5,6,7,8]. Nevertheless, finding a ‘one size fits all’ hemoglobin level below which blood transfusion is beneficial remains difficult [9,10]. Consequently, the question has been raised whether the decision for an RBCT should be based on the hemoglobin level alone [8].

Considering that mitochondria are the final destination for oxygen, mitochondrial oxygen tension (mitoPO_2_) measurement can theoretically give essential information about the adequacy of oxygen delivery capacity and the occurrence of cellular hypoxia. MitoPO_2_ can be measured non-invasively using the COMET device, which employs the protoporphyrin IX-triplet state lifetime technique [11]. The PpIX-TSLT is based on the delayed fluorescent properties of protoporphyrin IX. To stimulate the synthesis of endogenous mitochondrial PpIX through the conversion of 5-aminolevulinic acid (ALA), an ALA plaster (Alacare, Photonamic GmbH & Co.KG., Pinneberg, Germany) has to be applied to the skin for several hours. After subsequent excitation with light, the accumulated protoporphyrin IX emits a delayed red fluorescence. The delayed fluorescence lifetime is oxygen-dependent. Short lifetimes resemble high partial oxygen pressure, and long lifetimes resemble low oxygen pressure [12].

In prior animal experiments, we showed a correlation between a sudden drop in mitoPO_2_ and the critical hematocrit [13]. Based on these findings, we hypothesize that anemia-induced low mitochondrial oxygen tension does not improve after fluid administration but after a transfusion of erythrocytes (MOTIFATE). As clinical experience with the COMET monitor is limited, we first wanted to carry out a pilot study in a low-risk population under standardized measuring circumstances.

Whilst acute anemia is generally caused by acute (surgical) blood loss or hemolysis, chronic anemia is often a derivative of chronic disease [14]. The pathophysiology of chronic anemia in adults can be divided in three categories: a decreased production of RBCs (e.g., in myelodysplastic syndromes), an increased breakdown of RBCs (e.g., sickle cell anemia), or a loss of RBCs (e.g., heavy menstrual blood loss) [14]. To increase oxygen delivery and maintain an adequate oxygen supply in these patients, an RBC transfusion is given on a regular basis. Transfusion is often given in an elective setting during hospital daycare. Therefore, chronic anemia patients are considered a low-risk population for initiating transfusion-related research using the COMET monitor.

In light of the above, we conducted a pilot study in chronic anemia patients evaluating a clinical approach to test the MOTIFATE hypothesis. The primary endpoint of the study was mitoPO_2_ and its differential response to RBC transfusion compared to a fluid challenge. Furthermore, a secondary endpoint was to explore the clinical usability of the COMET monitor in blood transfusion treatments, especially the feasibility of performing measurements in an outpatient setting.

## 2. Materials and Methods

### 2.1. Study Design and Setting

This single-center pilot study was performed at the Erasmus Medical Center (Erasmus MC) in Rotterdam, the Netherlands. The protocol was approved by the Institutional Research Board (IRB) of Erasmus MC and registered in the Central Commission for research in humans register (NL55664.078.15). Written informed consent was obtained from all patients before any research activities.

Eligible patients were above 18 years of age and either presented with a low Hb level (<5.0 mmol/L) or clinical symptoms related to a low Hb according to the Dutch transfusion guidelines for chronic anemia patients [15]. Exclusion criteria consisted of intellectual disability or a medical history of porphyria, hemoglobinopathy, or heart or kidney failure with a fluid-restricted diet.

For participants who received an RBCT at the outpatient clinic, a 5-aminolevulinic acid (ALA) plaster (Alacare, Medac GmbH, Wedel, Germany) was mailed a few days in advance. It was recommended to clean and shave the skin before the ALA plaster was applied. 

The outpatients came for their regular hematologist visit in the Erasmus MC the next day. The hematologist determined the amount of transfused red blood cell units, which varied between 1 and 3 units and was based on the hemoglobin level in an arterial sample in the morning. One red blood cell unit consisted of 270 to 290 mL. If an RBCT was unnecessary, the patient went home after the ALA plaster was removed and replaced with a different, light-shielding plaster to protect the skin from sunlight. The protective plaster was allowed to be removed in the evening. 

For patients admitted to the hospital, Hb levels were checked daily to determine the need for an RBCT. If a patient was likely to receive an RBCT, informed consent was sought. The ALA plaster was applied around 10 p.m. on the day before the RBCT. If the RBCT was scheduled for the end of the day, the ALA plaster was applied at least 4 h before the intervention.

In addition, vital signs, such as blood pressure, temperature, and oxygen saturation were monitored. The RBCT started with 10 min slow infusion (60 mL/h) of the RBC unit to determine the occurrence of a transfusion reaction. Afterwards, the infusion rate was set to 300 mL/h, after which it took approximately 60 min to administer one RBC unit. In order to analyze the MOTIFATE hypothesis, before or after RBCT, a fluid challenge (FC) was given (500 mL 0.9% sodium chloride) to distinguish between a volume effect or an effect as a result of an increase in number of red blood cells. Both the erythrocytes and the saline infusion were not pre-warmed prior to infusion. 

Patients were allowed to participate two times in this study. The selection of patients who could participate twice was not pre-planned. Therefore, every subject who needed an RBCT on two different occasions during the study period could be assessed twice until the intended sample size was reached. The order of first receiving the RBCT or FC was decided using sealed envelopes, opened on the measurement day. If a patient participated multiple times, the order of transfusion of the RBCT and FC was reversed the second time. 

### 2.2. Recruitment and Consent

Patients in need of a blood transfusion were screened by the hematologist. If patients proved to be eligible for participation, their contact information was provided to the researcher. Subsequently, the researcher would telephone the patient, explain the study, and seek informed consent.

### 2.3. MitoPO_2_ Measurements 

After informed consent, a plaster of 5-aminolevulinic acid (ALA) (Alacare, Photonamic GmbH & Co.KG., Pinneberg, Germany) was either sent to the patient or applied by the researcher. The 5-aminolevulinic acid in the plaster was applied on the skin for at least 4 h to enhance the protoporphyrin IX (PpIX) concentration in the mitochondria. 

**Figure 1:**
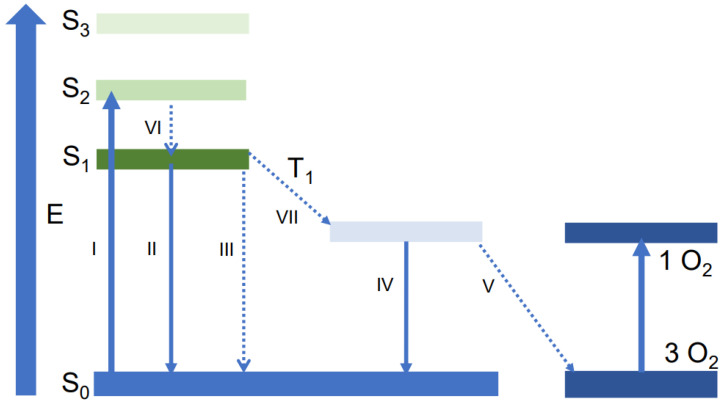
Jablonski diagram representing the states and transitions of porphyrins and oxygen, with S_0_, S_1_, S_2_, and S_3_ representing the ground state and three excited states of porphyrin. T_1_ equals the first excited triplet state of porphyrin, and 1 O_2_ and 3 O_2_ represent the excited singlet oxygen state and the triplet ground state of oxygen. I represents absorption, II is fluorescence, III are radiationless transmissions, IV phosphorescence, V energy transfer, VI represents internal conversion, and VII represents intersystem crossing.

PpIX is the final precursor of heme in the heme biosynthetic pathway and is synthesized in the mitochondria [16]. As the conversion of PpIX to heme is a rate-limiting step, the administration of ALA causes accumulation of PpIX inside the mitochondria. Delayed fluorescence can be detected after short excitation of PpIX within the red spectrum. The delayed fluorescence is the result of spontaneous relaxation with the emission of a photon. Oxygen is an effective quencher of this excited state. Energy is transferred to oxygen, and PpIX relaxes without the emission of a photon. The interaction between porphyrin and oxygen is visualized in a Jablonski diagram in Figure 1. The delayed fluorescence lifetime is related to mitoPO_2_ according to the Stern–Volmer equation:

$${\mathrm{PO}}_{2}=\frac{\frac{1}{\tau }-\frac{1}{\tau_{0}}}{k_{q}}$$

in which *τ* is the measured delayed fluorescence lifetime, *k_q_* is the quenching constant, and *τ_0_* is the lifetime at zero oxygen. The Stern–Volmer equation is valid for a homogenous oxygen distribution and after excitation with a pulse of light, of which the lifetime is much shorter than *τ.* In the case of a non-homogenous oxygen distribution inside the measurement volume, a reliable estimation of the average PO_2_ can be made using the rectangular distribution method (RDM) [17,18,19]. In-depth details of the method to calculate mitochondrial oxygen tension from PpIX delayed fluorescence can be found in Mik et al. [16], and the methodology behind the mitoPO_2_ measurements was previously described by Harms et al. [11,20].

**Figure 2:**
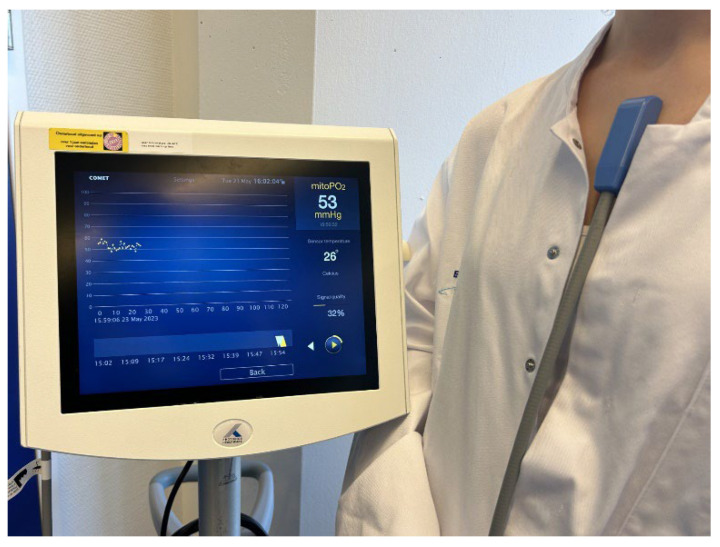
Clinical COMET system and skin sensor placed on the sternum of a volunteer. On the right side of the screen, mitoPO_2_ (mmHg), temperature (°C), and signal quality are displayed in numbers. On the left, a graph depicts mitoPO_2_ (mmHg) on the y-axis and time (min) on the x-axis.

After the introduction of the protoporphyrin IX-triplet state lifetime technique (PpIX-TSLT) in 2006, it has been extensively evaluated and calibrated in various tissues, including the liver, heart, and skin cells of study animals [16,21,22,23,24,25]. Further pre-clinical studies consisted of (calibration) studies in healthy volunteers, after which the focus shifted to clinical studies [20,26]. Since the introduction of the clinical COMET system in 2016, mitochondrial oxygenation has been analyzed in neonates and in neurosurgical, cardiothoracic surgery, COVID-19, and sepsis patients [27,28,29,30]. A depiction of the COMET system can be seen in Figure 2. 

### 2.4. Data Analysis 

The primary study parameter was mitoPO_2_ in kPa measured using the COMET device. The secondary study parameters included hemodynamic parameters and red blood cell age. As this was a pilot study, no a priori sample size was calculated. The IRB agreed on a pilot study comprising 16 inclusions per group, whereby patients could participate in both groups. 

Analysis of the data was split between patients measured twice (paired measurements) and patients measured once (unpaired measurements). Data were tested for normality using a Shapiro–Wilk test at every time point. Change in mean mitoPO_2_ from baseline after RBCT and saline infusion was analyzed using an ANOVA repeated measures test. The paired *t*-test was used for comparison of the primary and secondary parameters. To correct for multiple testing, a Bonferroni correction was applied for the alpha level. A linear regression model was used to assess the relation between delta mitoPO_2_ and the age of the given erythrocytes. Data analysis was performed using R statistics version 4.1.3 (R Foundation for Statistical Computing, Vienna, Austria) [31]. 

## 3. Results

**Figure 3:**
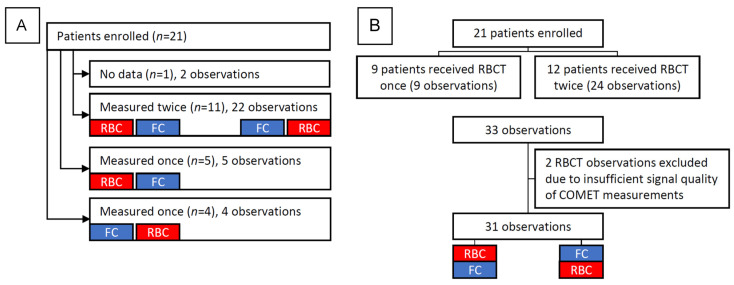
Flowcharts of patient enrollment (**A**) and study protocol (**B**). FC: fluid challenge, RBCT: red blood cell transfusion.

In total, 21 patients were enrolled (April 2016 till January 2018). Twelve of these patients were measured on two different occasions, whereby one time they first received an RBCT or an FC with saline and the second time vice versa. The other nine either received an RBCT or an FC. From the 33 observations, 2 measurements were not included in the analysis as a result of the insufficient signal quality of the mitoPO_2_ measurement. This resulted in 31 observations from 20 patients, as can be seen in the flowchart in Figure 3. In total, 50% of the patients were diagnosed with myelodysplastic syndrome or acute myeloid leukemia. The patient characteristics can be seen in Table 1. All patients remained hemodynamically stable throughout the measurements, as can be seen in Table 2. The median time between an RBCT and saline infusion was 6 min, with a minimum of 1 min and maximum of 22 min. The median time between a saline infusion and an RBCT was 7 min with a minimum of 1 min and a maximum of 39 min. Detailed information on the time in days between measurements for the patients that have been measured twice can be found in Table S1 in the Supplementary Materials. 

### 3.1. MitoPO_2_ after Red Blood Cell Transfusion and Fluid Challenge 

**Figure 4:**
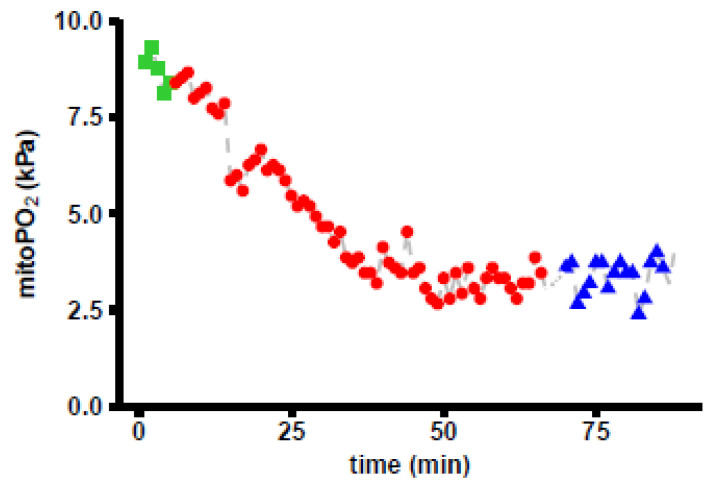
An example of mitochondrial oxygen tension (mitoPO_2_) measurements as measured using the COMET monitor (interval in min). The red dots represent the values during RBCT, and the blue triangles the values during a subsequent FC. The first five green squares are baseline measurements.

Figure 4 shows an example of the response in mitoPO_2_ after an RBCT followed by an FC. MitoPO_2_ was measured with an interval of 1 min. With respect to the overall response of the measurements, mitoPO_2_ dropped during the RBCT and did not change during the FC.

**Figure 5:**
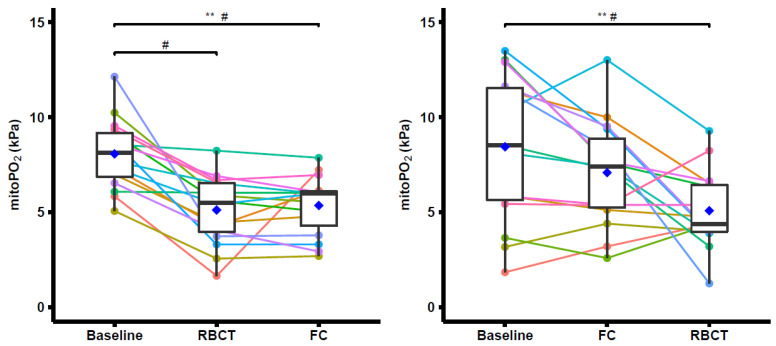
The mitochondrial oxygen tension (mitoPO_2_) in kPa for each group. The boxplot shows the median in the middle with hinges depicting the first and third quartiles (25 and 75% percentiles). Whiskers are the min–max values within 1.5 × interquartile range (IQR). The lines represent one data sequence per patient. # Paired *t*-test with Bonferroni correction, ** ANOVA repeated measures test, and blue diamond is the mean value.

Contrary to the MOTIFATE hypothesis, overall, in the group who received an RBCT first, the mean mitoPO_2_ measurements decreased significantly from baseline upon an RBCT (8.07 ± 1.82 kPa to 5.12 ± 1.78 kPa (*n* = 16; *p* < 0.05; mean ± standard deviation (SD)). After the subsequent saline infusion, an insignificant rise in the mean mitoPO_2_ to 5.36 ± 1.58 kPa was observed. In the group who started with the FC followed by the RBCT, the mean baseline mitoPO_2_ was 8.45 ± 3.92 kPa, which slightly decreased to 7.09 ± 2.80 kPa after the FC. The mean mitoPO_2_ was significantly reduced to 5.08 ± 2.04 kPa after an RBCT. The abovementioned results are displayed in Figure 5. Detailed sub comparisons with stratification of increasing and decreasing observations between timepoints can be found in the Figure S1 in the Supplementary Materials. 

### 3.2. Hemoglobin Level in Relation to mitoPO_2_

**Figure 6:**
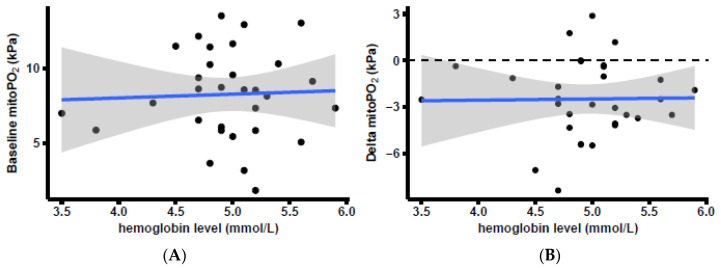
Correlation between mitochondrial oxygen tension (mitoPO_2_) and baseline hemoglobin level. (**A**) Linear regression model (blue line) of the relationship between baseline mitoPO_2_ (dots) and baseline hemoglobin level. (**B**) Delta mitoPO_2_ was calculated as the change in mitoPO_2_ value between baselines and after RBCT or the last part of both the saline infusion and RBCT, depending on the order in which the fluids were administered. The gray area is the standard error around the linear model.

No correlation could be found between either the baseline Hb level and baseline mitoPO_2_ (R^2^ = 0.002, intercept = 7.01 kPa, slope = 0.25 kPa, *p* = 0.82) or the baseline Hb level and delta mitoPO_2_ (R^2^ = 0.0002, intercept = −2.88 kPa, slope = 0.08 kPa, *p* = 0.94). Delta mitoPO_2_ was calculated as the change in the mitoPO_2_ value between either baseline and after an RBCT or the last part of both the FC and RBCT, depending on the order in which the fluids were administered. Figure 6 displays the corresponding graphs.

### 3.3. Delta mitoPO_2_ in Relation to Red Blood Cell Unit Age

The delta mitoPO_2_ in relation to red blood cell unit age could be analyzed in 25 of the 31 RBCT cases. In the measurements of the group who received RBCT first, delta mitoPO_2_ were constituted of the difference between the baseline mitoPO_2_ and mitoPO_2_ after an RBCT. In the measurements of the group who received a fluid challenge first, the delta mitoPO_2_ was based on the difference in mitoPO_2_ between mitoPO_2_ after an FC and after an RBCT.

**Figure 7:**
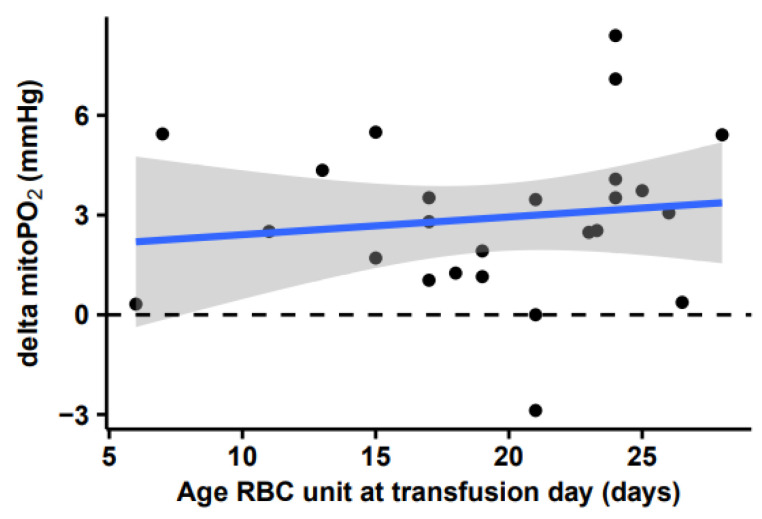
Correlation between change in mitochondrial oxygen tension (mitoPO_2_) red blood cell unit age. Linear regression model (blue line) of the relationship between delta mitoPO_2_ (dots) and age of the administered erythrocytes. Delta mitoPO_2_ was calculated as the change in mitoPO_2_ value between either baseline and after RBCT or the last part of both the FC and RBCT, depending on the order in which the fluids were administered. Gray area is the standard error around the linear model.

No correlation could be found between delta mitoPO_2_ and age for all given erythrocytes (R^2^ = 0.02, intercept = −1.87 ∆kPa decrease, slope = −0.05 ∆kPa/day, *p* = 0.54), as can be seen in Figure 7.

### 3.4. Skin Temperature during Red Blood Cell Transfusion and Fluid Challenge

**Figure 8:**
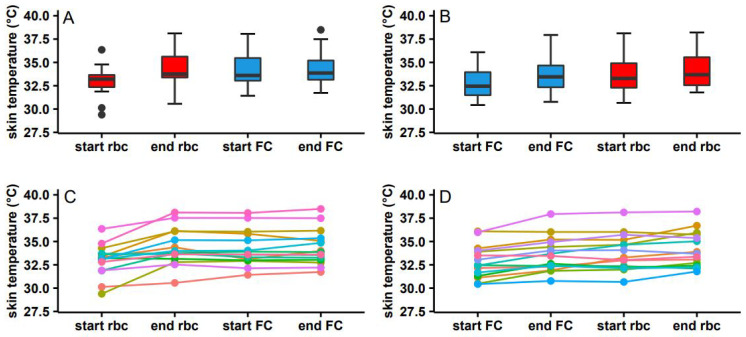
Change in skin temperature after red blood cell transfusion and fluid challenge. (**A**) Median skin temperature during the first sequence (RBCT followed by FC). (**B**) Median skin temperature during the second sequence (FC followed by RBCT). In panels (**C**,**D**), the individual start and end temperatures are presented using lines during the first (panel (**C**)) and second sequence (panel (**D**)). Within the boxplot, the median is represented in the middle with the hinges depicting the first and third quartiles (25 and 75% percentiles). The whiskers are the min–max values within 1.5 × IQR.

During RBCTs and FCs, skin temperature was measured as a proxy for probe temperature. No significant change in skin temperature was seen in the first sequence (an RBCT followed by an FC) between the start and end of an RBCT (*p* = 0.05) or the start of an FC and the end of an FC (*p* = 0.90). Neither was a significant change found in the second sequence (an FC followed by an RBCT) between the start and end of an FC (*p* = 0.32) nor between the start and end of an RBCT (*p* = 0.5897). These results are also depicted in Figure 8. 

## 4. Discussion

In this pilot study, we were able to continuously monitor mitoPO_2_ during RBCTs in chronic anemic patients in an outpatient setting. The most prominent effect of RBCTs on the median mitoPO_2_ was a decrease. This effect was not seen after infusions of 500 mL of saline. The overall decrease in mitoPO_2_ is an unexpected result. However, definite conclusions on the effect of RBCTs on mitochondrial oxygenation in comparison to FCs cannot be drawn based on the results of this pilot study, for the reason that the sample size is too small, and there is an absence of comparative measurements, such as using near infrared spectroscopy (NIRS).

In prior studies, the potential of monitoring mitoPO_2_ as an early detection method for adequate oxygen delivery capacity was highlighted. For instance, in the pre-clinical study by Römers et al. in 2016 [13], both skeletal muscle tissue oxygenation (StO_2_) using NIRS and mitoPO_2_ were measured in pigs during a hemodilution protocol. Whereas mitoPO_2_ showed a decrease preceding the decline in hemodynamic parameters and a rise in lactate, the NIRS values remained stable even though ischemia occurred [13]. The same phenomenon has been described in two clinical studies by Harms et al. [29]. During cardiac surgery, in contrast to mitoPO_2_**,** NIRS only decreased during extreme intraoperative events [29]. Furthermore, Harms et al. illustrated a case in which mitochondrial and microcirculatory parameters preceded a change in StO_2_ and hemodynamic parameters during ongoing blood loss [32].

Theoretically, mitoPO_2_ is the ultimate parameter for blood-transfusion-related research and transfusion medicine. Considering the physiology and mechanisms involved in oxygen transport and delivery to tissues, the decrease in mitoPO_2_ after RBCTs can be explained by several mechanisms. The first of these is that cell-free plasma hemoglobin may scavenge the important vasodilator nitric oxide (NO) and could decrease the microvascular flow due to vasoconstriction in the skin [33,34,35]. Moreover, the transfused RBCs could be less deformable [36,37] and aggregate in the microcirculation and reduce blood flow [36,38]. Microcirculatory flow could be additionally affected by an alteration in the plasma hematocrit ratio (the Fåhraeus–Lindqvist effect) [39,40].

Furthermore, 2,3-disphosphoglycerate (2,3-DPG) is a metabolic intermediate that allosterically affects hemoglobin oxygen affinity. After 10 days of storage, almost all red blood cells’ 2,3-DPG are depleted [41], and the oxyhemoglobin dissociation curve shifts to the left, increasing the hemoglobin affinity for O_2_. Consequently, less oxygen is released to the tissue. Our analysis revealed no correlation between mitoPO_2_ before and after blood transfusion and the age of given erythrocytes. This can be explained by the fact that only two of the given erythrocyte units were younger than 10 days, implying that in the majority of these units, 2,3-DPG was already depleted. The used research design was obviously unsuitable to investigate the effect of the depletion of 2,3-DPG on mitoPO_2_. A follow-up study focused on the age of the transfused erythrocytes and its effect on mitoPO_2_ is necessary to address this question.

Another possible explanation could be the concept of a critical Hb level. If the critical Hb level is reached, compensatory physiological responses to anemia are unable to maintain tissue oxygenation [42]. A further decline in Hb concentration will, therefore, lead to a decrease in the delivery of oxygen and oxygen consumption [43]. It has been suggested that the critical Hb level is not only patient- and disease-dependent but can also vary over time, as the compensatory responses to anemia may be affected by different comorbidities. As patients could also receive an RBCT if their Hb level was 5.0 mmol/L without clinical symptoms, it may be possible that the majority of the patients did not reach the critical Hb level. This could explain the seemingly contradictive effect of RBCTs on mitochondrial oxygenation in the few patients in our cohort, who demonstrated improved mitochondrial oxygenation after RBCTs [42]. Furthermore, it would also explain why no clinically significant changes were seen in mitoPO_2_ after saline infusion in our results. If the critical Hb level was reached in our patient population, it would be expected that mitoPO_2_ would decrease after saline infusion as a result of hemodilution. The suggestion that the individual critical Hb level could potentially be determined using mitoPO_2_ measurements provides opportunities for further research in both clinical and pre-clinical settings. The hypothesis in future studies would be that if the Hb level is low without a decreased mitoPO_2,_ the critical Hb level is not reached; therefore, an RBCT is not indicated. In the long term, the results of these studies may well lead to changes in the management of RBCTs, with, hopefully, a better patient outcome as a result.

The results of our study, wherein mitoPO_2_ did not improve upon RBCTs, are consistent with the systematic review by Nielsen et al., which was published in 2017 [44]. They reviewed all articles between 1947 and 2017 which measured the effect of RBCTs on tissue oxygenation or microcirculatory parameters in patients admitted to the intensive care unit (ICU) [44]. The used techniques included lactic acid measurement, gastric tonometry, NIRS, and sidestream dark field (SDF) imaging. The authors concluded that, generally, RBCTs did not improve tissue oxygenation or microcirculatory parameters in ICU patients with moderate anemia with a hemoglobin level between 4.34 and 6.21 mmol/L (7–10 g/dL) [44]. However, several of the studies also showed that patients with disturbances in tissue oxygenation or microcirculatory parameters did show improvement upon RBCTs. For these patients, the pre-transfusion baseline parameters for oxygenation and microcirculatory flow were correlated to the post-transfusion values, suggesting a difference in the benefit from RBCTs [44].

### Limitations

This study has several limitations. First, this is a pilot study with a sample size of 20 patients and 31 observations. Apart from the overall decrease in mitoPO_2_, an increase in mitoPO_2_ of more than 10% upon RBCTs was observed in four patients. As a result of the small sample size and the lack of post-RBCT parameters, however, we cannot clarify whether the difference between the increase and decrease in mitoPO_2_ is associated with an improvement in health complaints, microcirculatory parameters, hemodynamic parameters, or laboratory values. The aforementioned parameters were not included in the study protocol, as the focus of this pilot study was on mitoPO_2_ and its differential response to RBC transfusion compared to FCs and the clinical feasibility and approach of the measurements. Hence, a parallel analysis of standardized parameters, such as arterial PO_2,_ CO_2,_ serum lactate, or StO_2_, should be included in future follow-up studies.

Furthermore, the study population consisted of chronic anemia patients, as these patients regularly receive an RBCT at a planned appointment, which enables the placement of the ALA plaster 4 hr before the measurement. However, patients with chronic anemia have had time to physiologically adapt to compensate for the low Hb level and maintain necessary oxygen delivery [45]. This makes it difficult to translate our results to patients with acute anemia.

Lastly, we only measured mitoPO_2_ before, during, and shortly after RBCTs. The decrease in mitoPO_2_ could be a transient effect, after which patients experience the negative effects of RBCTs. This period could potentially be hours or days.

## 5. Conclusions

In this pilot study, we explored the use of an innovative technique that introduces a novel clinical parameter, mitoPO_2_, during blood transfusions in an ambulatory setting. The differential response of mitoPO_2_ to RBCTs in comparison to FCs was examined. We observed a decrease in mitochondrial oxygenation after RBCTs, which was not seen after saline infusions. The clinical significance of the decrease in mitoPO_2_ in terms of the clinical outcome remains to be elucidated. Our results should be validated in a larger, powered trial. Future research should preferably combine mitochondrial oxygenation and microvascular blood flow measurements and include additional outcome measures. In addition, it should be measured over the course of days to investigate if the effect is transient. To conclude, in combination with microvascular parameters, mitochondrial oxygenation measurements provide novel insight into the effect of RBCTs on cellular oxygenation. The results of this study encourage the exploration of mitoPO_2_ as a potential transfusion trigger or a means to personalized transfusion medicine.

## Figures and Tables

**Table 1:** Patient demographics and diagnoses. Values in mean ± standard deviation (SD).

Demographics	*n* = 20
Gender male (%)	*n =* 14 (70%)
Inpatient (%)	*n* = 7 (35%)
Age (years)	63 ± 10
Length (cm)	176 ± 10
Weight (kg)	80 ± 22
Diagnosis (%)	
Myelodysplastic syndrome	*n* = 5 (25%)
Acute myeloid leukemia	*n* = 5 (25%)
Acute lymphocytic leukemia	*n* = 4 (20%)
Primary myelofibrosis	*n* = 3 (15%)
Mantle cell lymphoma	*n* = 1 (5%)
Sideroblastic anemia	*n* = 1 (5%)
Aplastic anemia	*n* = 1 (5%)

**Table 2:** Values at baseline, end of red blood cell transfusion, and fluid challenge.

	Red Blood Cell Transfusion First				Fluid Challenge First			
	Baseline	RBCT	FC	*p*	Baseline	FC	RBCT	*p*
Hb (mmol/L) ^a^	5.0 ± 0.4	-	-		5.0 ± 0.4	-	-	NS
RBCT age (days) ^b^	-	19.1 ± 6.5	-		-	-	19.7 ± 5.4	NS
Body temp (°C) ^c,d^	36.9 36.7–37.1	37.0 36.7–37.5	36.8 36.6–37.2	NS	37.0 36.7–37.2	36.8 36.5–37.2	36.7 36.7–37.2	NS
SaPO_2_ (%) ^e^	96.8 ± 1.8	97.6 ± 1.6	97.9 ± 1.7	NS	96.6 ± 2.5	96.7 ± 2.5	97.1 ± 2.5	NS
Systolic NIBP (kPa) ^e^	16.2 ± 2.0	17.1 ± 3.3	17.1 ± 2.4	NS	16.2 ± 1.3	16.8 ± 1.7	17.1 ± 1.3	NS
Diastolic NIBP (kPa) ^e^	8.6 ± 1.1	9.6 ± 1.1	9.6 ± 1.4	NS	8.7 ± 1.0	8.9 ± 1.1	9.5 ± 0.9	NS
HR (beats/min) ^d^	69.5 66.5–88.3	64.0 61.8–87.5	63.0 62.0–83.5	NS	71.0 60.0–77.5	70.0 61.0–77.5	73.0 57.5–75.5	NS

FC: fluid challenge, Hb: hemoglobin, HR: heart rate, NS: not significant, NIBP: non-invasive blood pressure, RBCT: red blood cell transfusion, SaPO_2_: arterial saturation. ^a^ sample *t*-test, not paired. ^b^ RBCT → FC *n* = 13, FC → RBCT *n* = 12, sample t-test not paired. ^c^ body temperature, location tympanic. ^d^ Kruskal–Wallis test, paired true. ^e^ repeated measures ANOVA.

## Data Availability

Data are contained within the article.

## Supplementary Materials

The following supporting information can be downloaded at: https://www.mdpi.com/article/10.3390/biomedicines11071873/s1, Table S1: Time in days between measurements for the patients that have been measured twice; Figure S1: Sub comparisons with stratification increasing and decreasing observations between timepoints. (A) comparison Baseline and RBCT, (B) comparison RBCT and FC, (C) comparison Baseline and FC, (D) comparison between FC and RBCT.
