# Supplementary material for: Measuring Mitochondrial Oxygen Tension during Red Blood Cell Transfusion in Chronic Anemia Patients: A Pilot Study
Source: Biomedicines. 2023 Jun 30;11(7):1873. doi: 10.3390/biomedicines11071873 (PMC10376882; doi:10.3390/biomedicines11071873)
Supplement: Supplementary file 1 [file biomedicines-11-01873-s001.zip › biomedicines-1934899-supplementary.pdf]

Supplementary Materials

Table S1. Time in days between measurements for the patients that have been measured twice.

Subject Number	Time (days)
1	14
2	21
3	14
4	35
5	59
6	35
7	10
8	8
9	6
10	7
11	14

Subcohort Analysis

Stratified analysis of the mitoPO_2 data. Increase of decrease stratification was determined by the ratio. If the ratio was ≥ 1.0 it was an increasing observation, lower than 0.99 was considered a decreased observation. The following four sub comparisons were done.

- Group 1 RBCT values compared to the Baseline figure 1 A supplementary
- Group 1 FC values compared to RBCT figure 1 B supplementary
- Group 2 FC values compared to Baseline figure 1 C supplementary
- Group 2 RBCT values compared to FC figure 1 D supplementary

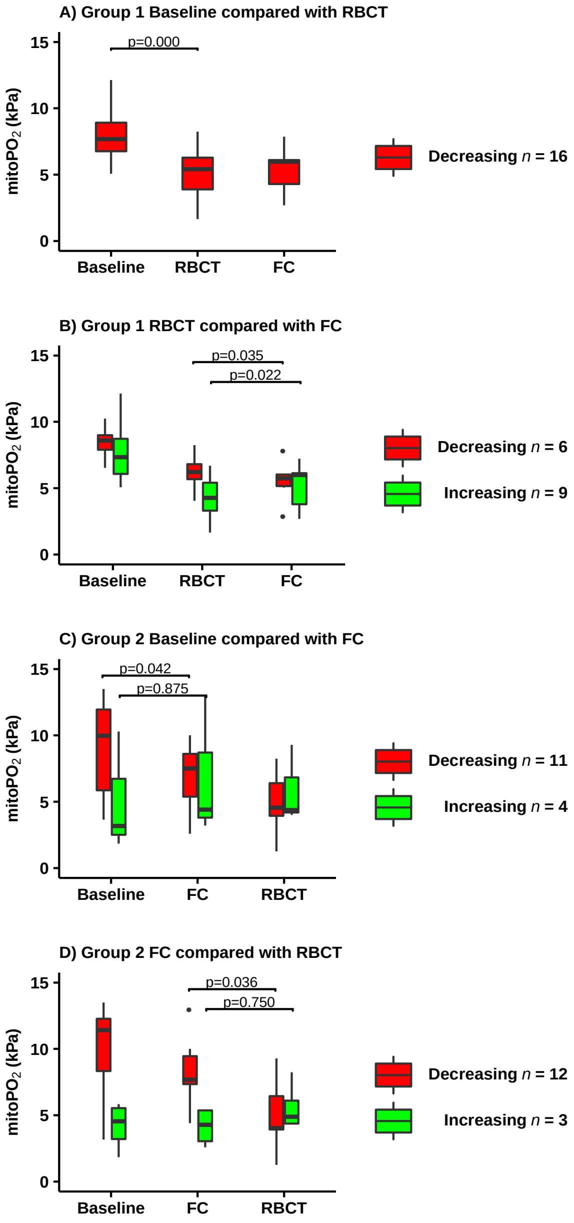

Figure S1. Sub comparisons with stratification increasing and decreasing observations between timepoints. (**A**) comparison Baseline and RBCT, (**B**) comparison RBCT and FC, (**C**) comparison Baseline and FC, (**D**) comparison between FC and RBCT.
